# Integrating oral PrEP delivery among African women in a large HIV endpoint‐driven clinical trial

**DOI:** 10.1002/jia2.25491

**Published:** 2020-05-12

**Authors:** Ivana Beesham, Julia D Welch, Renee Heffron, Melanie Pleaner, Lara Kidoguchi, Thesla Palanee‐Phillips, Khatija Ahmed, Deborah Baron, Elizabeth A Bukusi, Cheryl Louw, Timothy D Mastro, Jennifer Smit, Joanne R Batting, Mookho Malahleha, Veronique C Bailey, Mags Beksinska, Deborah Donnell, Jared M Baeten, the ECHO Trial Consortium, James Kiarie, Nelly R Mugo, Helen Rees, Jessica Justman, Zelda Nhlabatsi, Maricianah Onono, Linda‐Gail Bekker, Gonasagrie Nair, G Justus Hofmeyr, Mandisa Singata‐Madliki, Jennifer Smit, Sydney Sibiya, Jeffrey Stringer, Peter B Gichangi, Kate B Heller, Nomthandazo Mbandazayo, Charles S Morrison, Kavita Nanda, Caitlin W Scoville, Kathleen Shears, Petrus S Steyn, Douglas Taylor, Katherine K Thomas, Raesibe Agnes Pearl Selepe, Margaret Phiri Kasaro

**Affiliations:** ^1^ MatCH Research Unit (MRU) Faculty of Health Sciences University of the Witwatersrand Durban South Africa; ^2^ FHI 360 Durham NC USA; ^3^ University of Washington Seattle WA USA; ^4^ Wits Reproductive Health and HIV Institute Faculty of Health Sciences University of the Witwatersrand Johannesburg South Africa; ^5^ Setshaba Research Centre Tshwane South Africa; ^6^ Kenya Medical Research Institute (KEMRI) Nairobi Kenya; ^7^ Madibeng Centre for Research Brits South Africa; ^8^ Department of Family Medicine Faculty of Health Sciences University of Pretoria Pretoria South Africa; ^9^ Effective Care Research Unit (ECRU) Fort Hare and Eastern Cape Department of Health Universities of the Witwatersrand East London South Africa

**Keywords:** pre‐exposure prophylaxis, clinical trials, standard of care, women, HIV

## Abstract

**Introduction:**

Global guidelines emphasize the ethical obligation of investigators to help participants in HIV‐endpoint trials reduce HIV risk by offering an optimal HIV prevention package. Oral pre‐exposure prophylaxis (PrEP) has increasingly become part of state‐of‐the‐art HIV prevention. Here we describe the process of integrating oral PrEP delivery into the HIV prevention package of the Evidence for Contraceptive Options and HIV Outcomes (ECHO) Trial.

**Methods:**

ECHO was an open‐label randomized clinical trial that compared HIV incidence among women randomized to one of three effective contraceptives. In total, 7830 women aged 16 to 35 years from 12 sites in four African countries (Eswatini, Kenya, South Africa and Zambia) were enrolled and followed for 12 to 18 months, from 2015 to 2018. Part‐way through the course of the trial, oral PrEP was provided to study participants either off‐site via referral or on site via trained trial staff. PrEP uptake was compared between different contraceptive users using Chi‐squared tests or t‐tests. HIV seroincidence rates were compared between participants who never versus ever initiated PrEP using exact Poisson regression.

**Results:**

PrEP access in ECHO began through public availability in Kenya in May 2017 and was available at all sites by June 2018. When PrEP became available, 3626 (46.3%) eligible women were still in follow‐up in the study, and of these, 622 (17.2%) initiated PrEP. Women initiating PrEP were slightly older; more likely to be unmarried, not living with their partner, having multiple partners; and less likely to be earning their own income and receiving financial support from partners (all *p* < 0.05). PrEP initiation did not differ across study randomized groups (*p* = 0.7). Two‐thirds of PrEP users were continuing PrEP at study exit.

**Conclusions:**

There is a need for improved HIV prevention services in clinical trials with HIV endpoints, especially trials among African women. PrEP as a component of a comprehensive HIV prevention package provided to women in a large clinical trial is practical and feasible. Provision of PrEP within clinical trials with HIV outcomes should be standard of prevention.

## 1 Introduction

Within the context of clinical trials in which incident HIV infection is a primary study outcome, global guidelines emphasize an ethical imperative to assist participants reduce HIV risk by offering state‐of‐the‐art prevention packages [1, 2, 3, 4]. Guidelines do not detail a specific set of recommended HIV prevention interventions, and best practices for HIV prevention have evolved over time. Risk‐reduction counseling, HIV counseling and testing, and provision of condoms have been commonly provided, and more recent trials have included testing and treatment for sexually transmitted infections (STIs), HIV and STI testing of partners, voluntary medical male circumcision for HIV‐uninfected men, and initiation of antiretroviral therapy (ART) for partners living with HIV [1]. Importantly, the components of prevention packages should reflect community and stakeholder involvement in making decisions about what constitutes an optimal HIV standard of prevention, tailored to the context of each locality, trial and changing expectations about effective prevention services.

In 2015, the World Health Organization (WHO) issued a global recommendation that antiretroviral‐based pre‐exposure prophylaxis (PrEP) become a component of combination HIV prevention [5]. Many countries have since incorporated oral PrEP into their national HIV prevention strategies [6, 7, 8]. Incorporating PrEP into standard of care HIV prevention services for trials with HIV outcomes has emerged as an important component of trial design and operational plans [9]. How to incorporate new prevention interventions into the standard prevention package of clinical trials comes with ethical, operational and scientific complexities, and this area is currently an active topic of ethical, advocacy and research discussion [4, 10].

The Evidence for Contraceptive Options and HIV Outcomes (ECHO) Trial was an open‐label, randomized clinical trial testing the relative effects of three effective contraceptive methods on HIV acquisition risk, with incident HIV as the primary study endpoint [11]. The ECHO Trial protocol called for a comprehensive HIV prevention package to be provided to all participants, including PrEP as it became a part of national prevention policies in the host countries and as national recommendations for PrEP emerged during the trial period. Despite the provision of a comprehensive HIV prevention package to women during the ECHO Trial, the HIV incidence was 3.8 per 100 women‐years [12]. Here, we describe the process undertaken by the trial team to incorporate oral PrEP delivery as part of the ECHO Trial HIV prevention package.

## 2 Methods

### 2.1 Design and follow‐up

The ECHO Trial was conducted between December 2015 and October 2018 and followed 7829 women at twelve study sites in four African countries (Eswatini, Kenya, South Africa and Zambia) (Clinicaltrials.gov NCT02550067). Women desiring contraception were randomized in a 1:1:1 ratio to depot medroxyprogesterone acetate by intramuscular injection (DMPA‐IM), a copper intrauterine device (IUD), or a levonorgestrel (LNG) implant. Follow‐up was quarterly for up to 18 months. Incident HIV infection was the primary study endpoint. Rapid HIV testing was done at baseline, quarterly and if clinically indicated or requested by a participant [11]. Participants signed written informed consent. Approval was obtained from Research Ethics Committees (RECs) associated with each trial site and the Protection of Human Subjects Committee [12].

The trial’s community engagement was informed by Good Participatory Practice (GPP) guidelines including developing a GPP plan at each site that detailed community engagement activities and establishing a platform to discuss technical support for GPP [2]. A Global Community Advisory Group (GCAG) was established to provide a forum for civil society, advocates and other stakeholders to engage with the ECHO team about the trial conduct and discuss how broader issues related to HIV prevention and contraceptive use might impact the trial and policies globally.

### 2.2 HIV prevention in the ECHO Trial and PrEP availability

The ECHO Trial protocol specified that all participants would be provided a standard HIV prevention package at all visits that included risk reduction counseling, provision of HIV testing and screening, STI testing and treatment, offering condoms, partner HIV counseling and testing, and counseling on ART for HIV risk reduction, and referral for ART in HIV serodiscordant couples [12]. When ECHO launched participant recruitment in 2015, PrEP was not yet considered standard of care in the host countries. A key consideration in the decision to provide PrEP was accessing PrEP through local means available which would be more sustainable over time. The protocol allowed for women to use PrEP at any time, and for the offering of PrEP on site if it was incorporated into national HIV prevention policies.

The four ECHO Trial host countries incorporated PrEP into national policies in different ways and times. In South Africa, HIV prevention policies were updated in 2016 to include PrEP for all persons with substantial HIV risk but public PrEP programmes were initially restricted to select clinics and demonstration projects for sex workers, eventually expanding to include men who have sex with men, serodiscordant couples and adolescent girls and young women [8]. Kenya introduced PrEP guidance in 2016 and national rollout in May 2017 with universal access to all people with substantial HIV risk [13]. In Eswatini and Zambia, PrEP pilots in government‐sponsored demonstration projects began in 2017. In November 2017, the South African Medical Research Council (SAMRC) held a stakeholder meeting, attended by members of the ECHO GCAG, about the evolving PrEP standard of care and issued a recommendation that PrEP should be made available to research participants in clinical trials with HIV endpoints in South Africa [14]. With the support of the GCAG, the ECHO Trial team explored the opportunity to make PrEP available on site at all twelve ECHO sites through trial funds. For women from Kenya and Zambia, it was determined that there was sustainable access to PrEP at referral centres that would continue post‐trial. For women from Eswatini, the research site was not part of the country’s demonstration project programme, and the site used referral to relevant sites for PrEP access. For the nine sites in South Africa, the ECHO team proceeded to take the steps necessary to make PrEP available at the research sites.

PrEP was made available to ECHO Trial participants through two mechanisms: (1) on site as part of the HIV prevention package and (2) off‐site via referral.

### 2.3 Process of PrEP integration at the South African ECHO Trial sites

All nine South African ECHO sites began offering PrEP between March and June 2018 following relevant consultation, training and determination of operational readiness. The SAMRC guidance recognized that discussion and input from the local community stakeholders and RECs was essential to ensure meaningful implementation of PrEP [14], and these groups were included in the decision‐making process. The decision to provide PrEP on a voluntary basis was supported by the overseeing REC’s.

Training of site staff was conducted during the first quarter of 2018 at each trial site. For the training, components were adapted from the South African standardized PrEP training package, and the South African National Department of Health (NDoH) PrEP Guidelines [15]. In addition, field‐tested material developed by the Southern African HIV Clinicians Society, the NDoH, and an Optimizing Prevention Technology Introduction on Schedule were used [16]. Training was coordinated with the South African NDoH guidelines to ensure alignment with the national plan. The training also incorporated provider sensitization, the provision of PrEP as part of an optimal combination HIV prevention package for ECHO Trial participants, the importance of participants exploring their own risk profile and risk reduction options, and adherence to and effective use of PrEP. An important component of the training included communication with participants about post study access to PrEP. Where possible, participants who started PrEP on site would be referred to demonstration projects and public facilities to access PrEP at study exit. The entire site staff teams were included in the training, including cadres representative of the pharmacists, counselors, and outreach, administrative, clinical and quality assurance staff. Additional targeted training was designed for the different staff roles, including in‐depth training on informational resources, materials and referrals with each site’s community and outreach team. Separate educational workshops and presentations on PrEP were also conducted with each research site’s Community Advisory Boards (CABs).

Operational aspects of PrEP readiness focused on costs, commodities and logistical aspects of PrEP delivery: South African national PrEP guidelines include laboratory testing (HIV, pregnancy, creatinine and hepatitis B virus testing) prior to initiation; the PrEP drug (generic emtricitabine/tenofovir disoproxil fumarate) needed to be procured and distributed; and hepatitis B vaccination would be offered. PrEP visits were scheduled according to South African national guidelines, and visits were generally aligned where possible with ECHO Trial study visits. PrEP was offered to participants as part of the HIV prevention package on a voluntary basis in women who were eligible under national PrEP clinical guidelines. PrEP was only offered to participants who were still in active follow‐up and had at least one month remaining in the trial. Participants who had initiated PrEP on site and desired to continue PrEP were given a three‐month supply of PrEP at their final trial visit and referred to facilities providing PrEP where available.

### 2.4 Process of PrEP integration at the non‐South African sites

Participants from sites in Eswatini, Kenya and Zambia were counselled on PrEP by trial staff, and those who were interested were referred to facilities off‐site. Site investigators and community liaison officers established linkages to off‐site facilities providing PrEP where participants could receive PrEP, such as government‐approved demonstration sites.

### 2.5 PrEP data collection

At each study visit, participants were asked a standardized question about whether she had used PrEP since the last study visit and the dates of PrEP use were recorded. Because PrEP was not a research component of the trial, additional data specific to PrEP were not captured (e.g. reasons for refusal, side effects, laboratory test results specific to PrEP, adherence).

### 2.6 Data analysis

#### 2.6.1 Access to PrEP

For each participant, we defined the date PrEP became available as the earlier of (1) the date when their ECHO site began offering PrEP (South African sites only) or PrEP became widely available per national programme rollout (Kenyan site only), or (2) the self‐reported date of PrEP initiation at an off‐site facility (Eswatini and Zambia). Participants who could have initiated PrEP were defined as those who either met the on‐site criteria for being offered PrEP (i.e. attended a study visit after PrEP was available, had at least one month remaining in study follow‐up, were HIV‐uninfected, and at sites in South Africa and Zambia, were not pregnant or breastfeeding), or who were ascertained to have received PrEP off‐site. PrEP was not recommended for pregnant and breastfeeding women in South Africa and Zambia as per country guidelines at the time.

#### 2.6.2 PrEP use

PrEP medications and dates of use were obtained through on‐site records of PrEP use using case report forms that were completed by study clinicians on dispensing PrEP and via participant self‐report. We defined PrEP use as continuing at study exit if the participant had previously initiated PrEP, attended her final scheduled follow‐up visit, and at her final scheduled follow‐up visit either received PrEP on site or self‐reported ongoing use of PrEP received off‐site.

### 2.7 Statistical analysis

Comparisons of characteristics between PrEP groups were assessed using Chi‐squared tests (or Fisher’s exact tests for uncommon characteristics) for categorical variables and t‐tests for continuous variables. HIV seroincidence comparisons between participants who never versus ever initiated PrEP were modeled using exact Poisson regression and included covariates that were both significantly different between the never versus ever PrEP users in Table 1.

**Table 1:** Participant characteristics and association with PrEP initiation

Participant Characteristics: N (%) or median (IQR)				
Characteristic	Had access to but did not initiate PrEP (N = 3004)		Ever initiated PrEP (N = 622)	*p*‐value
Baseline characteristics				
Age group (years)				<0.0001
16 to 17	57 (1.9%)		4 (0.6%)	
18 to 20	820 (27.3%)		129 (20.7%)	
21 to 24	1082 (36.0%)		218 (35.0%)	
25 to 30	815 (27.1%)		200 (32.2%)	
31 to 35	230 (7.7%)		71 (11.4%)	
Never married	1999 (66.5%)		558 (89.7%)	<0.0001
Lives with partner	1194 (39.7%)		134 (21.5%)	<0.0001
Partner provides financial/material support	2188 (72.8%)		277 (44.5%)	<0.0001
Education				<0.0001
None	27 (0.9%)		2 (0.3%)	
Primary (any)	530 (17.6%)		27 (4.3%)	
Secondary (any)	1974 (65.7%)		496 (79.7%)	
Post‐secondary (any)	473 (15.7%)		97 (15.6%)	
Earns own income	800 (26.6%)		107 (17.2%)	<0.0001
Number of partners in the past three months				0.006
0	12 (0.4%)		0 (0%)	
1	2840 (94.6%)		573 (92.1%)	
≥2	151 (5.0%)		49 (7.9%)	
Condom use with last vaginal sex				<0.0001
Yes	1331 (44.3%)		317 (51.0%)	
No, had condomless sex	1486 (49.5%)		294 (47.3%)	
No, no vaginal sex	174 (5.8%)		11 (1.8%)	
No, no partner	12 (0.4%)		0 (0%)	
Chlamydia trachomatis	475 (15.8%)		127 (20.4%)	0.005
Neisseria gonorrhoeae	121 (4.0%)		33 (5.3%)	0.15
Voice modified risk score category^a^				<0.0001
0 to 4	1719 (57.6%)		224 (36.1%)	
5 to 8	1267 (42.4%)		396 (63.9%)	
Characteristics at PrEP access date (if did not initiate PrEP) or PrEP initiation date (if ever initiated PrEP)				
Number of partners in the past three months				<0.0001
0	92 (3.1%)	12 (1.9%)		
1	2815 (93.8%)	561 (90.6%)		
≥2	93 (3.1%)	46 (7.4%)		
Condom use with last vaginal sex				0.009
Yes	1177 (39.2%)	262 (42.3%)		
No, had condomless sex	1550 (51.7%)	326 (52.7%)		
No, no vaginal sex	180 (6.0%)	19 (3.1%)		
No, no partner	92 (3.1%)	12 (1.9%)		

^a^ The VOICE risk score is composed of demographic and behavioural characteristics, with higher values reflecting greater expected HIV incidence [17].

## 3 Results

**Figure 1:**
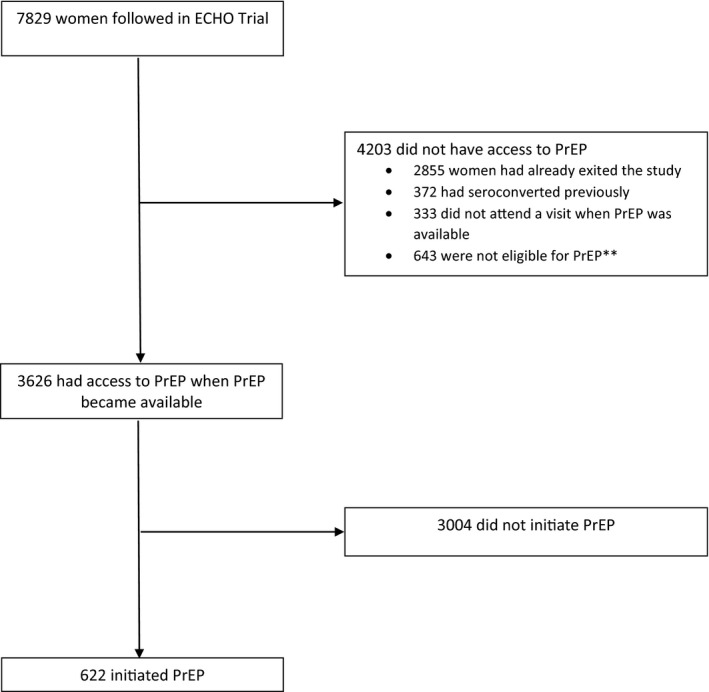
PrEP eligibility and initiation in the ECHO Trial. **Of the 643 women who were not eligible, 128 had less than 28 days of follow up at sites that were providing PrEP on‐site and 515 were either pregnant (26) or breastfeeding (489) at all follow up visits when PrEP was available (in sites other than Kenya and Eswatini). PrEP access was defined as beginning in May 2017 for the site in Kenya, in November 2017 for the site in Zambia, in December 2017 for the site in Eswatini, and between March 2018 and June 2018 for the nine sites in South Africa. ECHO, Evidence for Contraceptive Options and HIV Outcomes; PrEP, pre‐exposure prophylaxis.

Of the 7829 women followed in the ECHO Trial, 3626 (46.3%) had access to PrEP prior to exiting the study and 622 (17.2% of those with access) initiated PrEP (Figure 1) Of the 622, 11 initiated in Eswatini (11 of 363 with access, 3.0%), 45 in Kenya (45 of 889, 5.1%), 559 in South Africa (559 of 2069, 27.0%) and 7 in Zambia (7 of 305, 2.3%). For those in South Africa, 49 women had initiated PrEP prior to on‐site provision at the South African sites.

The median age of women who initiated PrEP was 24 years (IQR 21 to 27). Women who initiated PrEP compared to those who did not were slightly older, more likely to be unmarried, not living with their partner, having more than one partner and less likely to be earning their own income and receiving financial support from partners (Table 1). Women who initiated PrEP also had a higher VOICE modified risk score (median = 5, IQR 4 to 7) [17] compared to those who did not initiate PrEP (median = 4, IQR 2 to 6). The prevalence of *Chlamydia trachomatis* at enrolment was higher in women who initiated PrEP (*p* = 0.005). Condomless sex at the time of PrEP access was high both in women who did not initiate PrEP as well as women who initiated PrEP with just over half of women in both groups reporting that the last vaginal sex act was without a condom. Most women reported having one sexual partner at the time of PrEP access (>90%), and 46 (7.4%) women who initiated PrEP reported two or more partners at the visit when PrEP was initiated.

There was no significant difference in uptake of PrEP by randomized contraceptive group with 188 women randomized to DMPA‐IM, 216 randomized to copper IUD and 218 randomized to LNG implant initiating PrEP (*p* = 0.75). Findings were similar when analysed by contraceptive method in use at the time of PrEP initiation: 194 DMPA‐IM, 189 copper IUD and 212 LNG implant (*p* = 0.68). Among women who initiated PrEP, the median duration of PrEP use was 85 days prior to study exit (IQR 40 to 96), and two‐thirds of women (66.6%) were continuing PrEP at study exit.

There were 37 HIV seroconversions among women who had access to PrEP but did not initiate PrEP and 2 seroconversions among women who initiated PrEP (HIV incidence 2.4 versus 1.0 per 100 person‐years, IRR 0.35, 95% CI 0.04 to 1.38). Of the two women who seroconverted after starting PrEP, one had stopped PrEP approximately two months prior to seroconversion. The other woman reported continuous PrEP use for 28 days, followed by a two‐day break in use, and then continuous use for 52 days until the date of seroconversion. She tested negative on rapid HIV testing both at baseline when initiating PrEP and 30 days later at her first follow‐up visit post PrEP initiation.

## 4 Discussion

The ECHO Trial provided PrEP as a component of a comprehensive HIV prevention package. In other trials with HIV as the primary endpoint, HIV prevention packages provided to participants have included risk‐reduction counselling, free condoms, referral for or provision of male medical circumcision, testing and treatment of STIs, and counselling and referral for post‐exposure prophylaxis. In recent years, substantial discussion has focused on the ethical, logistical and scientific considerations regarding incorporation of oral PrEP into clinical trials with HIV as the primary endpoint, as part of a standard prevention package or an active comparator for new PrEP agents [4, 10]. The ECHO Trial represents one of the first trials with incident HIV as a primary study outcome to successfully incorporate PrEP into a standard HIV prevention package, provided on site, and could be used as a model for future trials as PrEP becomes part of standard prevention services.

Almost one in five women with access to PrEP initiated it, and women who initiated PrEP had characteristics suggesting an increased risk for acquiring HIV. Other HIV prevention trials have found similar characteristics among participants, such as a high prevalence of *C. trachomatis* and inconsistent condom use [18, 19]. Recent studies have found good uptake of PrEP among young women in Africa [20].

For South Africa, there was a substantial difference in the uptake of PrEP prior to the on‐site provision with only 49 women initiating PrEP prior to on‐site provision but> 500 initiating after PrEP was available on site. In addition, nearly 90% of those who initiated PrEP were from the South African sites. Together, these suggest that on‐site provision of PrEP might be important for stimulating greater uptake in trial settings like this.

There were similar ratios of PrEP uptake among women by randomized contraceptive method and current contraceptive method in use. Theoretically, it was possible that women using DMPA‐IM would be more likely to initiate PrEP due to concerns about the possible increased risk of acquiring HIV with DMPA‐IM use, as women were extensively counselled about this potential risk when enrolling into the study and during follow‐up as a result of updating WHO guidance about the change of progestogen‐only injectables among women at high risk for HIV from a WHO MEC category 1 to category 2 which occurred during the course of the trial [21]. However, we did not find greater uptake of PrEP in women using DMPA‐IM compared to the other contraceptive methods.

We found that two women who had initiated PrEP had seroconverted compared to 37 women who did not initiate PrEP. Of the two women who had seroconverted, one of these had stopped PrEP two months prior to HIV detection. Our trial was not designed to collect detailed data about PrEP use and directly assess PrEP efficacy, which has been demonstrated in more rigorous and well‐powered studies already [6].

The well‐being of trial participants is the highest priority for clinical investigators and every effort should be made to help prevent HIV infection in clinical trials with HIV endpoints. It is likely that the provision of PrEP for persons at high risk for HIV will reduce the rate of HIV seroconversions, therefore affecting the study power in HIV endpoint trials. In ECHO, PrEP was offered late into the trial and thus would not be expected to have a significant impact on the total number of HIV infections in the trial. Nevertheless, this potential limitation should not be a barrier to providing oral PrEP in future trials. It is still possible to obtain study outcomes but it might take a longer period of time to achieve these; and the provision of PrEP should be factored into study power calculations. Novel trial designs and analyses are being considered in response to effective HIV prevention provision to all participants [10]. Not all women who are offered PrEP will initiate it, and not all women who initiate PrEP will continue using it or remain adherent to achieve optimal levels of protection. For example, in the recent HPTN 082 study, despite an uptake of PrEP in 95%, detectable plasma tenofovir concentrations declined from 65% at three months to 25% at twelve months [22].

A key limitation of this analysis is that even though the ECHO Trial encouraged, facilitated and documented the use of PrEP as part of a combination HIV prevention package, the trial was not explicitly designed to study PrEP, therefore data on PrEP adherence, reasons for choosing to use PrEP or not, and reasons for discontinuing PrEP were not collected. One of the considerations for the provision of PrEP was participants’ post‐study access to PrEP. Participants in the ECHO Trial who commenced PrEP on site in the South African sites were counselled about the availability of PrEP and post‐study access to PrEP, and where possible, participants were referred to demonstration projects and other sites that were providing PrEP. Post‐study access is important not just for PrEP but also for other trial benefits such as contraception and other forms of HIV prevention. We hope that the positive response to the uptake of PrEP in this trial will provide supportive information to governments, policymakers and regulatory bodies that can lead to policies improving public access to PrEP.

Women in Africa and in particularly South Africa are at high risk of acquiring HIV [23]. In the ECHO Trial, HIV incidence was 3.8 per 100 women‐years overall and 4.5 per 100 women‐years for the nine South African sites [12]. Similar HIV incidence rates have been found in both the FEM‐PrEP and VOICE trials [18, 19]. WHO recommends that offering PrEP should be a priority for populations with an HIV incidence of about 3 per 100 person‐years or higher [5]. HIV prevention trials are conducted among communities and populations at high HIV risk in order to be able to evaluate the efficacy of new interventions. Thus, PrEP provision in such trials must be a priority. PrEP may not be acceptable for all women but it can be offered as an option that is part of an optimal HIV prevention package and women can be given a choice about whether they want to initiate PrEP or not, after being provided with the necessary information and counselling to make an informed decision.

The successful delivery of PrEP in ECHO demonstrates the feasibility of including PrEP in the standard prevention package offered within clinical trials. Barriers to access diminished when PrEP was offered on site, and the number of accepters was high. Our experience shows that with coordinated planning, research sites can quickly train staff, obtain PrEP medication and initiate PrEP services; these lessons are applicable to other clinical trials with HIV as a primary endpoint. The high proportion of women who accepted PrEP during ECHO demonstrates there is a demand for PrEP by this population. Moreover ECHO’s integration of PrEP within a study primarily focused on contraceptive services suggest these services are an ideal entry point for HIV prevention.

## 5 Conclusions

There was a high HIV incidence in the ECHO Trial and women who initiated PrEP had characteristics suggesting higher HIV risk. The provision of PrEP as part of HIV prevention standard of care in the ECHO Trial was logistically feasible, and it was possible to provide PrEP in a manner that was aligned with host countries’ policies and guidelines. Other clinical trials with incident HIV as a primary study outcome can and should plan to offer PrEP as part of a standard HIV prevention package.

## Competing interests

None were declared.

## Authors’ Contributions

IB and JDW drafted the initial manuscript. LK and DD analysed the data. IB, JDW, RH, MP, LK, TP, KA, DB, EAB, CL, TDM, JS, JRB, MM, VCB, MB, DD and JMB contributed to the integration of PrEP in the ECHO Trial, and all authors have read the manuscript, provided critical review and approved the final manuscript.

## Authors’ Information

*ECHO Trial Consortium:

Management Committee:

Jared M Baeten (University of Washington, Seattle, WA, USA), James Kiarie (WHO, Geneva, Switzerland), Timothy D Mastro (FHI 360, Durham, NC, USA), Nelly R Mugo (Kenya Medical Research Institute, Nairobi, Kenya & University of Washington, Seattle, WA, USA), Helen Rees (Wits Reproductive Health and HIV Institute, Johannesburg, South Africa).

Study Site Principal Investigators:

Eswatini, Manzini: Jessica Justman, Zelda Nhlabatsi (Family Life Association of Eswatini & ICAP at Columbia University, New York, NY, USA). Kenya, Kisumu: Elizabeth A Bukusi, Maricianah Onono (Kenya Medical Research Institute, Nairobi, Kenya). South Africa, Brits: Cheryl Louw (Madibeng Centre for Research). South Africa, Cape Town: Linda‐Gail Bekker, Gonasagrie Nair (University of Cape Town & Desmond Tutu HIV Centre). South Africa, Durban: Mags Beksinska, Jennifer Smit (MatCH Research Unit (MRU), Faculty of Health Sciences, University of the Witwatersrand). South Africa, East London: G Justus Hofmeyr, Mandisa Singata‐Madliki (University of Fort Hare & University of the Witwatersrand). South Africa, Edendale: Jennifer Smit (MatCH Research Unit (MRU), Faculty of Health Sciences, University of the Witwatersrand). South Africa, Johannesburg: Thesla Palanee‐Phillips (Wits Reproductive Health and HIV Institute, Faculty of Health Sciences, University of the Witwatersrand). South Africa, Klerksdorp: Raesibe Agnes Pearl Selepe (The Aurum Institute). South Africa, Ladysmith: Sydney Sibiya (Qhakaza Mbokodo Research Clinic). South Africa, Soshanguve: Khatija Ahmed (Setshaba Research Centre). Zambia, Lusaka: Margaret Phiri Kasaro, Jeffrey Stringer (UNC Global Projects Zambia & University of North Carolina at Chapel Hill, Chapel Hill, NC, USA).

Other members of the ECHO Trial Consortium:

Deborah Baron (Wits Reproductive Health and HIV Institute, Faculty of Health Sciences, University of the Witwatersrand, Johannesburg, South Africa), Deborah Donnell (University of Washington and Fred Hutchinson Cancer Research Center, Seattle, WA, USA), Peter B Gichangi (International Centre for Reproductive Health – Kenya & Technical University of Mombasa, Mombasa, Kenya), Kate B Heller (University of Washington, Seattle, WA, USA), Nomthandazo Mbandazayo (Wits Reproductive Health and HIV Institute, Johannesburg, South Africa), Charles S Morrison (FHI 360, Durham, NC, USA), Kavita Nanda (FHI 360, Durham, NC, USA), Melanie Pleaner (Wits Reproductive Health and HIV Institute, Faculty of Health Sciences, University of the Witwatersrand, Johannesburg, South Africa), Caitlin W Scoville (University of Washington, Seattle, WA, USA), Kathleen Shears (FHI 360, Washington, DC, USA), Petrus S Steyn (WHO, Geneva, Switzerland), Douglas Taylor (FHI 360, Durham, NC, USA), Katherine K Thomas (University of Washington, Seattle, WA, USA), Julia D Welch (FHI 360, Durham, NC, USA).

Data management was done by DF/Net, Inc. (Seattle, USA); laboratory support was done by Clinical Laboratory Services (CLS), a division of the Wits Health Consortium (WHC) of the University of Witwatersrand’s School of Pathology. (Johannesburg, South Africa).
